# A new species of perennial *Bromus* (Bromeae, Poaceae) from the Iberian Peninsula

**DOI:** 10.3897/phytokeys.121.32588

**Published:** 2019-04-24

**Authors:** Carmen Acedo, Félix Llamas

**Affiliations:** 1 Research Group Taxonomy and Conservation of Biodiversity TaCoBi, Department of Biodiversity and Environment Management, University of León, E-24071. León, Spain University of León León Spain

**Keywords:** *
Bromea
*, *
Bromus subg. Festucoides
*, *Bromuserectus* complex, *
Bromus picoeuropeanus
*, Cantabrian range, Identification Key, New species, Poaceae, Spain, Taxonomy

## Abstract

During a survey of the genus *Bromus* for the ongoing Flora Iberica, *B.picoeuropeanus***sp. nov.**, a new orophilous species of perennial *Bromus* from Picos de Europa National Park, was found, and it is described and illustrated here. This new species belongs to the *Bromuserectus* complex and differs from the other perennial species of this group occurring in the Iberian Peninsula in its well-developed rhizome, the small innovation leaves and all peduncles and branches shorter than the spikelets. *B.picoeuropeanus* grows on calcareous stony soils associated with dry places. We provide a description and illustrations of the new species and an identification key for the most related European perennial species belonging to the complex.

## Introduction

The genus *Bromus* L. is the only representative of the tribe Bromeae in Southwest Europe. The genus comprises perennial and annual species, and both life forms are distributed throughout the genus. *Bromus* has a characteristic fleshy appendage above the style insertion, which is pubescent at its apex and persistent in the caryopsis. The taxonomy of this genus is considered troublesome and has been the subject of numerous regional taxonomic revisions (e.g. Smith and Sales 1993; Acedo and Llamas 1999; Saarela 2008), and the main subject of several studies (e.g. Smith 1972; Acedo and Llamas 2001; Saarela et al. 2007; Oja and Zimmermann 2010; Alonso et al. 2014; Alonso 2015). It includes more than 170 species mainly in the Northern hemisphere (Acedo and Llamas 2001; Saarela et al. 2014).

The last revision of the genus *Bromus* L. in the Iberian Peninsula (Acedo and Llamas 1999) included a detailed discussion of its taxonomic history, morphology, anatomy, distribution and other relevant information for the region. Some twenty-five species of *Bromus* occur in the Iberian Peninsula. The Iberian monograph includes only two alien species, *B.catharticus* Vahl and *B.inermis* Leyss., and recently another weedy species was found: *B.sitchensis* Trin. (Acedo, unpublished data), native to northwestern North America. Some taxonomic studies (Oganesian 2004; Tzvelev 1976), the Euro+Med List of Plants (Valdés and Scholz 2006; 2009) or the Flora of Italy (Pignatti et al. 2017), and Flora of Russia (Fedorov 1999) treated the Old World perennial *Bromus* at the generic level as *Bromopsis* (Dumort.) Fourr. However, other authors argue it must be treated as Bromussect.Bromopsis Dumort. (Saarela 2008; Naderi and Rahiminejad 2015: 243) or subgenus Festucoides (Stebbins 1981; Acedo and Llamas 1999; Alonso, Llamas, Pimentel, and Acedo unpublished data) based on karyological and morphological data, and phylogenetic relationships suggested by molecular nuclear and plastid data regions. This study follows the proposal to split the genus *Bromus* into subgenera.

The European perennials belong to Bromussubg.Festucoides (Coss. & Durieu) Hackel, which is not monophyletic in its current circumscription (Saarela et al. 2007; Alonso 2015). It comprises between sixty and seventy perennial species, including caespitose or rhizomatous plants, ranging in height from twenty to more than one hundred and fifty centimeters, and growing in diverse terrestrial habitats such as forest, hedges and pastures, etc. In Europe, only a few species are associated with temperate forests (Smith 1981; Acedo and Llamas 1999). In this subgenus, the cross-section of the leaf blade is characterized by soft ribs and numerous primary vascular bundles bound by wide and complete sclerenchyma beams. The narrow spikelets with close parallel sides have glumes which are 1–3(5)-nerved (Acedo and Llamas 1999). The presence or absence of a developed long rhizome is considered an important diagnostic character within the perennial *Bromus*. Its presence is diagnostic for several European and West Asian species of *Bromus* as several authors pointed out in their identification keys (i.e. Tzvelev 1976; Smith 1980; Pignatti et al. 2017). In addition, the presence of a developed rhizome and the presence of tuberous basal internodes are diagnostic in North African taxa belonging to this group (Maire et al. 1955). Several European perennial taxa are caespitose and lack rhizomes (Smith 1980; Pignatti et al. 2017). The European perennials form a morphologically heterogeneous group, including taxa with broad distributions as well as narrow endemisms that are grouped within several complexes of cytologically and morphologically similar species (e.g. the *Bromuserectus* complex and the *Bromusramosus* complex). The *Bromuserectus* complex includes perennial species, with old sheaths remaining intact or decaying into parallel fibres, inflorescences with some long branches and/or pedicels, and spikelets erect (Smith 1980, Acedo et al. 2009; Pignatti et al. 2017). The taxonomy of some species or groups was studied in different regions e.g. “*Bromuserectus* Group” in Slovenia (Bačič and Jogan 2001) or the species *B.erectus* along the Cantabrian range and Pyrenean mountains in the Iberian Peninsula (Acedo et al. 2009). The *Bromuserectus* complex includes several endemics and probably some taxa remain undescribed, due to the lack of a global taxonomic revision.

The main objectives of this study are to describe a new species, to differentiate it from its relatives, and to characterize this new taxon.

## Materials and methods

Several specimens of perennial *Bromus* were collected during a survey of the genus *Bromus* for the ongoing *Flora Iberica* project. This material was confirmed as a new species after a careful study and comparison with material deposited at JBAG, LEB, FCO, MA, MAF, JACA, SALA, SANT, VIT, representing the full distribution and variability of *B.erectus* Huds. from the Iberian Peninsula as well as specimens belonging to related species (*B.condensatus* Steud., *B.stenophyllus* Link, *B.transsilvanicus* Steud.) and other perennial European species (*B.biebersteinii* Roem. & Schult., *B.moellendorffianus* (Asch. & Graebn.) Hayek, *B.moesiacus* Velen., *B.pannonicus* Kumm. & Sendtn., *B.riparius* Rhemann, *B.tomentellus* Boiss.). In addition, material from several important European Herbaria: C, K, FI, MSNM was studied. We also studied specimens and photographs of types and original material in B, P, and K, including the type specimen of *B.erectus* (Llamas and Acedo 2019, in press). Herbarium acronyms follow Thiers (2018+ continuously updated).

Specimen locality data were recorded in the field or via geo-referencing. We assessed the preliminary conservation status of the new species using our field knowledge, applying the IUCN (2017) criteria and performing a GeoCat analysis (Bachman et al. 2011). The extent of occurrence (EOO) and the area of occupancy (AOO) were estimated using GeoCat. For AOO calculation, a 2 km cell width was used. The information and measurements of the new and closely related species were taken from live and dried herbarium specimens, and from field data. The taxonomic treatment of the genus *Bromus* follows Acedo and Llamas (1999). Measurements and data for the diagnostic characters comparing the new species and *B.erectus* are presented in Table 1, and an identification key is provided to facilitate differentiation from the related European species.

**Table 1.** Summary of the main taxonomic traits that differentiate *Bromuspicoeuropeanus* Acedo & Llamas from *B.erectus* Huds.

	*B.picoeuropeanus* Acedo & Llamas	*B.erectus* Huds.
Habit	Loosely tufted	Densely caespitose
Rhizome presence	Rhizomatous	None, or inconspicuous rhizome, caespitose
Height	Up to 40 cm	60–130 cm
Basal and cauline leaf blade	Flat, basal similar to the cauline, 2–3.5 mm wide	The basal narrower (c.1 mm) and longer than cauline (2–3 mm wide)
Ligule	Truncated or rounded, 0.5- 1 mm	Blunt, (0.5)1–2 mm
Panicle in well-developed specimens	Contracted, 3–5(–8) cm length	Spreading, 10–20 cm length
Spikelet number	4–8(–11)	(8)20-30
Spikelet length	16–21 (–25) mm	(15–)20–35(50) mm
Branch length	Shorter than spikelet	Several branches equal to or longer than spikelet
Lower glume length	6–7 mm	7–12 mm
Upper glume length	7–9 mm	(8–)9–14(–15) mm
Fertile lemma length	9–11(–12) mm	(9–)10–15 (–18) mm
Palea length	Similar to lemma	Similar to lemma
Awn length	(2.5–)4.5–5 mm	2.5–6(8) mm
Florets number	(4–)5–7	(5–)7–9
Anther length	3.5–4.5 mm	4.5–8 mm
Caryopsis	Thickened, inrolled shorter than palea 8–9 mm	Thin and almost flat, similar to palea in length

## Results – taxonomic treatment

### Bromus picoeuropeanus

Taxon classificationPlantaePoalesPoaceae

Acedo & Llamas sp. nov.

urn:lsid:ipni.org:names:60478523-2

#### Type.

Spain. Cantabria: Macizo Oriental de Picos de Europa, Vegas de Ándara: Fuente de la Escalera. 43°12.42'N, 4°42.20'W, [WGS-84], on limestone dry rocky sites, moving by gelifraction, 1869 m alt., 31 August 2011; *C. Acedo, A. Alonso & F. Llamas* CA247.4 (Holotype LEB 121814).

#### Diagnosis.

*Bromuspicoeuropeanus* differs from *B.erectus* Huds. (Table 1) in having shorter habit; longer creeping rhizomes; non-cauline leaf blades short and never reaching the inflorescence, flat and similar to the cauline leaves; ligule truncated or round up to 1 mm; panicle 3–5(8) cm, contracted and smaller, with few spikelets, up to 11; all branches shorter than the spikelets; caryopsis thickened, inrolled or plicate, 8–9 mm, shorter than palea. *B.picoeuropeanus* also differs in its preference for stony soils.

#### Description.

**Figure 1.**
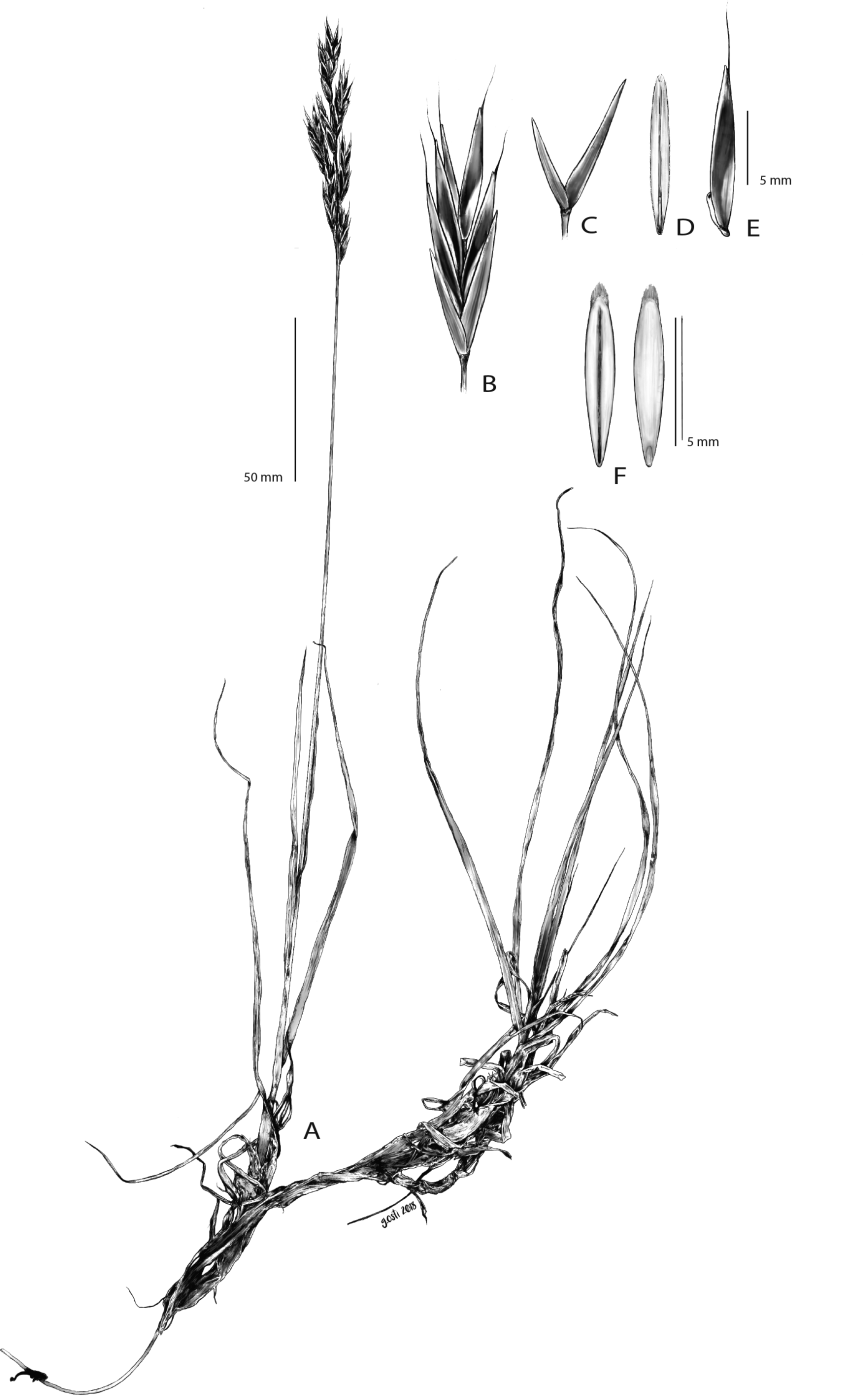
***Bromuspicoeuropeanus***. **A** Habit showing the developed rhizomes, the short basal leaves, and the contracted inflorescence **B** Spikelet with unequal glabrous glumes and five florets **C** Glumes **D** Palea in adaxial view, showing the two adaxial wings **E** Lemma in lateral view (drawn from the holotype) **F** Caryopsis in adaxial and abaxial view (drawn from LEB 121815). Drawings by Ms. Giulia Osti, 2018.

Perennial plant with long rhizomes 3–5(7) cm, loosely tufted. Flowering culms up to 40 cm. Culms channeled and glabrous, with glabrous nodes. Extravaginal innovation leaves with short blades, similar to the cauline leaves. Leaf sheaths of cauline leaves glabrous. Old basal leaf sheaths persistent, investing the culm base. Blade of cauline leaves 9–13 cm × 2–3 mm, tapering gradually towards the apex. Ligule membranous and glabrous, short, 0.5–1 mm, apex truncated or rounded, ± lacerated. Panicle 3–4(–8) × 2–3 cm, erect, lax, contracted, with 4–8(11) spikelets, branches slender. Scale of the lower node leaf-like, c. 4 mm, glabrous. Pedicels scabrid with fine antrorse teeth. All branches and pedicels shorter than spikelets. Spikelets 16–22(–25) × 3–5 mm, with two unequal glumes and 4–5(–7) fertile florets, imbricate when young, in maturity the florets slighted separated. Lower florets bisexual, 9–11(–12) mm, oblong, scaberulous toward the apex; upper floret male or sterile, 5–6 mm, lanceolate, glabrous, similar in color and texture to the lower florets. Lower glume 1–veined, narrow, 6–7 mm. Upper glume 3-veined, 7–9 mm. Lemma glabrous, lanceolate, section slightly keeled, 9–11(–12) mm (excluding the awns), 3–5-veined. Apex of the lemma slightly emarginate (sinus approximately 0.1 mm); margin rounded. Awn short, (2.5–) 3–4 (–5) mm, up to 1/3 the lemma length, fine and straight, inserted 1–1.5 mm below the apex. Rhachilla 2–3 mm, scabrid with very fine antrorse teeth. Callus short, glabrous and rounded. Palea linear-lanceolate of similar size or slightly shorter than the lemma, 8–11 × 1–2 mm, with aculeolate keels; wings nearly as wide as the palea body, with smooth border. Lodicules 2, lanceolate to oblong, glabrous, 0.5–1.5 mm long. Stamens 3, with anthers 3.5–4.5 mm long. Caryopsis elliptic, enrolled or plicate at maturity, 7–8 mm, shorter than palea (Fig. 1).

#### Phenology.

Flowering July – August. Fruiting August – September.

#### Distribution and habitat.

**Figure 2.**
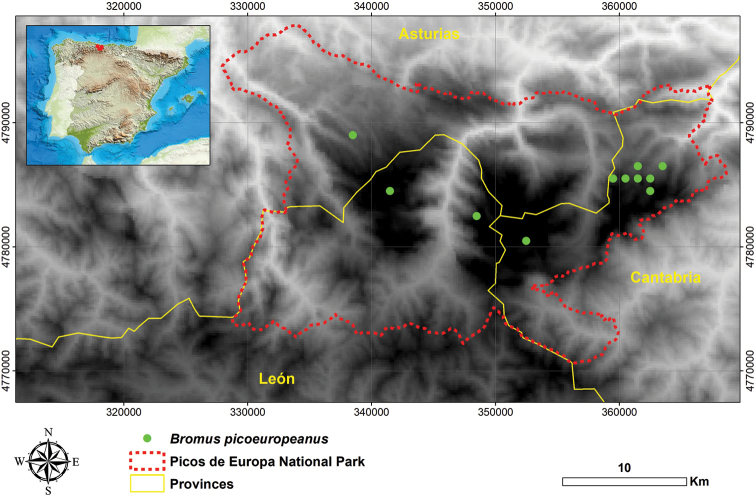
Distribution map representing all the known locations of *Bromuspicoeuropeanus*.

*Bromuspicoeuropeanus* is endemic to the Iberian Peninsula and occurs in Spain, distributed through the Northern Mountains of the Cantabrian Range (Fig. 2). We collected it in several localities of Picos de Europa National Park, growing in dry rocky areas of limestone moving by gelifraction, and on stony areas at an altitude of 1600–2200 m.

#### Conservation status.

*Bromuspicoeuropeanus* occurs within the Picos de Europa National Park. Although the IUCN (2017) criterion B thresholds (EOO = 111.51 km^2^; AOO = 40.0 km^2^) suggest a different category [EN], the species has been evaluated DD (Data Deficient), because further study is needed to assess the risk.

#### Etymology.

The specific epithet is a reference to the Spanish National Park Picos de Europa, where it was collected.

## Discussion

Some thirty perennial species belonging to the genus *Bromus* occur in the Euro+Med area (Valdés and Scholz 2006; 2009). Five perennials live in the Iberian Peninsula (Smith 1980; Acedo and Llamas 1999) including the cultivated and naturalized *Bromusinermis* Leyss. extensively cultivated all over the world, the weeds *B.catharticus* Vahl, and *B.sitchensis* Trin., and the native perennial species, *B.benekenii* (Lange) Trim., *B.ramosus* Huds., and *B.erectus*. This last species is the only one known in the Iberian Peninsula belonging to the *B.erectus* complex until now.

The *Bromuserectus* complex is differentiated by its persistent basal sheaths remaining intact or decaying into parallel fibers, and the non-cauline basal leaves typically longer and narrower than the cauline leaves. Its inflorescence is lax, spread or contracted, with erect branches and pedicels, more or less developed, but some of the pedicels longer than the spikelet. The multiflorous spikelet is supported by two subequal or unequal glumes with 1–5 nerves (Smith 1981; Acedo and Llamas 1999; Cope and Gray 2009; Pignatti et al. 2017). Other perennial species have old basal sheaths forming a reticulum as *B.moesiacus* Vell. or *B.riparius*. The complex lacks auricles as opposed to other perennial species having long lanceolate auricles (e.g. *B.biebersteinii*) or diminished auricles (e.g. *B.stenostachyus* Boiss.).

**Figure 3.**
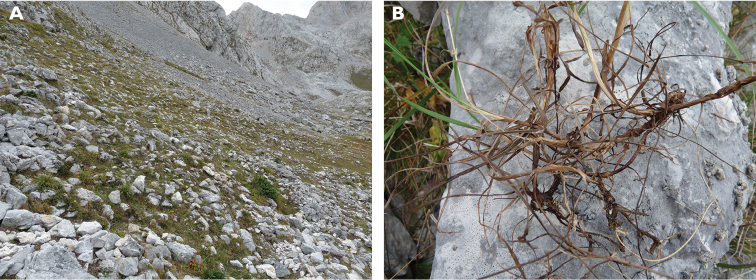
**A** The preferred habitat of *Bromuspicoeuropeanus* in stony and unstable soils, c. 1900 m elevation, where it prefers steep slopes and stony grassland, and disappears when the slope decreases or the pasture becomes denser **B** Detail of the basal part of culms showing the long rhizomes and flat cauline leaves.

The morphological traits of *Bromuspicoeuropeanus* suggest it must be classified within the *Bromuserectus* complex. Among the Iberian perennials, the specimens of *B.picoeuropeanus* are morphologically more similar to the widespread *B.erectus*. The presence of a developed rhizome 3–5(7) cm long (Fig. 1, 3) is a major difference with the remainder of the perennial Iberian taxa. This trait is relevant also in the differentiation of other perennial species such as the nemoral *B.benekenii* and *B.ramosus* (Cope and Gray 2009). There are other European species related to *B.erectus* which have rhizomes: *B.moellendorffianus*, *B.pannonicus*, *B.riparius*, or *B.tomentellus*. All of them, however, have very short rhizomes. The shoot leaf blades of *B.picoeuropeanus* are shorter (Fig. 1) than those of *B.erectus*, which has long and narrow leaves frequently reaching the inflorescence. Leaf hairs are very rare in *B.picoeuropeanus*, even more scarce than in *B.erectus*. In detailed descriptions of *B.erectus*, there is a large range of variability in other characters that are not useful for separating taxa: for instance, some floras (Pignatti et al. 2017) recognized *Bromuslongiflorus* Willd. ex Spreng. as having long spikelets with 11–13 florets, which is a variation of *B.erectus*. We have observed that the number of florets is a variable character in several species (Acedo and Llamas 1999; Acedo et al. 2009), and the floret number can be sometimes twice or more than its usual number.

While *Bromuserectus* has a wide distribution in most of Europe (except on the Scandinavian peninsula and the adjacent Northeastern countries), *B.picoeuropeanus* is known only in the National Park of the Picos de Europa, a small territory of the Cantabrian range. *B.picoeuropeanus* differs also in its ecological behavior from all the native Iberian perennial species. *B.benekenii* and *B.ramosus* occur in nemoral habitats and *B.erectus* mainly in mesophyllous meadows. *B.picoeuropeanus*, by contrast, occurs in stony and rocky places.

## Key to the perennial European species similar to *Bromuspicoeuropeanus*

**Table:** 

1	Plants rhizomatous, laxly caespitose; shoot and cauline leaf-blades similar in width, leaves glabrous, scabrid or distinctly pilose	**2**
–	Plants without rhizomes or stolons, or inconspicuously rhizomatous, tufted, caespitose; cauline leaf blades wider than the lower, with scattered long (up to 1mm) and patent hairs	**6**
2	Leaves flat or slightly involute	**3**
–	Leaves conduplicate, or setaceous-conduplicate	**4**
3	Rhizome long; lemma 9–11(–12) mm, awn 3–5mm; caryopsis slightly thickened, inrolled at maturity, shorter than palea; leaves not auriculate; culms up to 40 cm	*** B. picoeuropeanus ***
–	Rhizome short, forming distinct clumps; lemma long 11–14(–20) mm, awn 5–8 mm; caryopsis almost flat, similar in length to the palea; lower leaves shortly auriculate; culms 50–90(–120) cm	*** B. riparius ***
4	Leaves and sheaths with long greyish hairs; spikelets 15–20(–25) mm	*** B. pannonicus ***
–	Indumentum of leaves different; spikelets 15–25(–35) mm, awn similar in length to the lemma or slightly shorter	**5**
5	Leaf sheaths and blades tomentose, covered by dense short and sparse long hairs; lemma 12–18 mm, awn 11–17mm	*** B. tomentellus ***
–	Leaf blades and sheaths glabrous, scarcely scabrid on the veins or distinct pilose, not tomentose; lemma 8–10(–15) mm, awn 7–9 mm	*** B. moellendorffianus ***
6	Leaf sheaths lanate-pubescent, with long and tangled hairs; the lower sheaths fibrous; panicle denser; lemma 8–9 mm	*** B. condensatus ***
–	Leaf sheaths not lanate-pubescent, the lower sheaths persistent, remaining intact when dead; panicle lax, lemma > 9 mm	**7**
7	Glumes subequal, florets strongly overlapped, for ¾ of their length by the floret below; panicle spread	*** B. erectus ***
–	Glumes markedly unequal; florets only slightly overlapped by the apex of the floret below	**8**
8	Lower sheaths densely pubescent; lemma 13–18 mm, longer than the upper glume; awn up to one half of lemma length	*** B. stenophyllus ***
–	Sheaths glabrous or with few scattered long (c. 1 mm) hairs; lemma short, c. 10 mm, similar in length to the upper glume; awn similar to lemma length	*** B. transsilvanicus ***

## Other *Bromuspicoeuropeanus* specimens examined

**Asturias: Vegarredonda**, 43°14.44'N, 4°59.42'W, 1983, July 28, limestone, 1800 m alt., *H.S.Nava* s.n. (FCO 14203). **Cantabria: Canal de Jenduda**, 43°9.88'N, 4°48.88'W, 20 July 2008, 1810 m alt., *C.Acedo* & *F.Llamas* (v.v.); **Canal de San Carlos**, 43°12.70'N, 4°41.56'W, 6 August 1983, 1718 m alt., *H.S.Nava* s.n. (FCO 14201); **Canto La Concha**, 43°13.13'N, 4°40.81'W, 6 August 1983, 1660 m alt., *H.S.Nava* s.n. (FCO 14196); **Majada de la Redondal**, 43°12.52'N, 4°43.41'W, 3 August 1983, 1800 m alt., *H.S.Nava* s.n. (FCO 14200); **Mancondiu**, 43°12.96'N, 4°42.47'W, 6 August 1983, 1900 m alt., *H.S.Nava* s.n. (FCO 14199); **Pozo de Ándara**, 43°12.67'N, 4°43.80'W, 3 August 1983, 1730 m alt., *H.S.Nava* s.n. (FCO 14202); **Samelar**, 43°12.54'N, 4°41.90'W, 1 August 2007, 1700 m alt., *C.Acedo & F.Llamas* (v.v.); **Vegas de Ándara**: Fuente de la Escalera, 43°12.42'N, 4°42.20'W, 31 August 2011, 1869 m alt., *C.Acedo, A.Alonso & F.Llamas* CA247.1 (LEB121812); ibidem CA247.2 (LEB 121810); ibidem CA247.3 (LEB 121811); ibidem CA247.4, (LEB 121814); ibidem CA247.5 (LEB 121813); 50m East of the Fuente de la Escalera, 43°12.43'N, 4°41.99'W, 1 October 2017, 1886 m alt., *V.Ezquerra & C. Frey s.n.* (LEB 121815); Camino hacia Fuente de la Escalera, 43°12.46'N, 4°42.01'W, 1 October 2017, 1860, *V.Ezquerra & C.Frey* (v.v.), sink holes, 43°12.66'N, 4°42.25'W, 1 October 2017, 1789, *V.Ezquerra & C.Frey* (v.v.), road margin, 43°12.70'N, 4°42.24'W, 1 October 2017, 1787 m alt., *V.Ezquerra & C.Frey* (v.v.). **León. Carbayal**, 43°11.82'N, 4°57.11'W, 7 July 1983, 1800 m alt., *H.S.Nava* s.n.(FCO 14198); **Las Colladinas**, 43°10.84'N, 4°51.76'W, 22 July 1983, 2170 m alt., *H.S.Nava s.n.* (FCO 14197).

## Supplementary Material

XML Treatment for Bromus picoeuropeanus
